# Models for Training in Pediatric Otologic Surgery: A Systematic Review

**DOI:** 10.3390/children13040562

**Published:** 2026-04-18

**Authors:** Elena Carlotto, Serena Cirillo, Stefania Marconi, Silvia Pisani, Mirko Bertozzi, Cesare Chiapperini, Simone Mauramati, Marco Benazzo, Pietro Canzi

**Affiliations:** 1 Department of Clinical, Surgical, Diagnostic and Pediatric Sciences, University of Pavia, 27100 Pavia, Italy; elena.carlotto@unipv.it (E.C.); mirko.bertozzi@unipv.it (M.B.);; 2 Department of Otorhinolaryngology, Fondazione IRCCS Policlinico San Matteo, University of Pavia, 27100 Pavia, Italy; 3 Department of Civil Engineering and Architecture, University of Pavia, 27100 Pavia, Italy; 4 Department of Drug Sciences, University of Pavia, 27100 Pavia, Italy; 5 Pediatric Surgery Unit, Fondazione IRCCS Policlinico San Matteo, 27100 Pavia, Italy

**Keywords:** pediatric otology, temporal bone, surgical simulation, 3D printing, virtual reality, surgical training, virtual reality surgical simulation

## Abstract

**Highlights:**

**What are the main findings?**

- The limited availability of conventional training opportunities in pediatric otosurgery has driven increasing interest in simulation-based approaches.
- We provide a comprehensive synthesis of the current literature on models used for pediatric otologic surgical training.
- Three-dimensional printed temporal bone models are the most widely adopted simulation modality, followed by virtual reality environments.

**What are the implications of the main findings?**

- The high anatomical fidelity of 3D-printed models makes them especially valuable for both surgical skill acquisition and patient-specific preoperative planning.
- VR models enable reproducible and adaptable training across procedures of varying complexity.

**Abstract:**

**Background/Objectives**: Temporal bone surgery in children is technically challenging due to their smaller anatomical structures, developmental differences, and the closer proximity of critical neurovascular structures. The limited availability of conventional training materials and pediatric cadaveric specimens has led to greater enthusiasm for simulation-based methods. The aim of this systematic review was to identify existing otologic simulation models and evaluate their anatomical accuracy, teaching effectiveness, and supporting evidence. **Methods**: In accordance with PRISMA guidelines, the PubMed, Embase, Scopus, and Cochrane Library databases were searched for studies reporting simulation tools for pediatric otologic surgery. Articles describing three-dimensional printed (3DP) models, virtual reality (VR) platforms, cadaver specimens, and animal models were included. Studies focusing on children and providing educational outcomes were selected. The extracted data were synthetized and analytically discussed. **Results**: Thirteen studies met the inclusion criteria: nine on 3DP models and four on VR environments. No research involving cadavers or animals was identified. 3DP models exhibited consistent anatomical accuracy and notable educational advantages. Five studies used surveys for their evaluations, and three relied on expert observer assessments. The studies including validation analyses showed a high correlation between printed models and computed tomography (CT) images. VR systems supported anatomical reconstruction and segmentation tasks, as well as guided simulation exercises. However, most of the research consisted of feasibility studies with limited participant groups. **Conclusions**: Simulation-based training with 3DP and VR models could be ethical and accurate methods for obtaining relevant skills in pediatric otologic surgery. The reviewed data suggest that these tools may be suitable as a first-line step within an integrated, multimodal training pathway prior to direct patient contact.

## 1. Introduction

Temporal bone surgery in children presents technical challenges compared with adult otologic surgery. Indeed, children have smaller anatomical dimensions, a developing mastoid, a relatively exposed facial nerve, and a thin calvarium [1]. These factors reduce the operative field and increase the risk of iatrogenic injury. Traditional surgical training based on supervised operating room experience may not adequately prepare surgeons for high-risk pediatric cases. Furthermore, access to pediatric cadaveric temporal bones is extremely limited due to ethical, legal, and cultural constraints. This makes the integration of alternative educational techniques in training programs desirable. To address these kinds of limitations, the literature has demonstrated a growing interest in various simulation-based training models, such as three-dimensional printed (3DP) and virtual reality (VR) tools, in many fields [2,3,4]. These tools allow surgical skills to be developed in a safe environment with expert feedback prior to treating living patients [5]. However, evidence regarding their diffusion, technical characteristics, clinical relevance, and validation within pediatric otologic training remains fragmented across heterogeneous studies.

The objective of this systematic review is to synthesize the current literature on simulation-based models used for pediatric otologic surgical training, including 3DP models, VR systems, pediatric cadaveric specimens, and animal models.

Specifically, we aim to (1) identify and categorize the types of models described in the literature; (2) assess reported outcomes, including anatomical fidelity, educational effectiveness, and feasibility; and (3) map existing gaps to facilitate future research and guide the development of pediatric-specific training tools.

## 2. Materials and Methods

### 2.1. Search Strategy

A systematic review was performed following the Preferred Reporting Items for Systematic Reviews and Meta-Analyses (PRISMA) guidelines throughout the research process.

The data sources accessed during this study included PubMed, Embase, The Cochrane Library, and Scopus databases. The search was tailored according to the requirements of each database. Search strategies combined controlled vocabulary terms (MeSH/Emtree) and free-text keywords related to the temporal bone, otologic surgery, pediatric populations, simulation tools (VR, 3DP), and cadaveric or animal models, using Boolean operators. All search strings are listed in Supplementary Materials. The reference lists of the relevant articles were screened as a secondary search. Two authors (S.C. and C.C.) independently conducted the search of indexed English literature to minimize potential selection bias. The literature review was performed in August 2025 and updated in November 2025.

### 2.2. Eligibility Criteria

Articles of interest were original studies on pediatric temporal bone surgical training using, alternatively, 3DP, VR, cadaveric, or animal-based models. They had to focus on pediatric-specific models or models that were explicitly used for pediatric cases. Additionally, they had to report educational, anatomical, or technical outcomes. Studies referring to both endoscopic and microscopic temporal bone surgery were included. Only articles published in English were considered.

The exclusion criteria were non-English articles, non-surgical studies (e.g., audiologic physiology, electrophysiology, and pure imaging studies without training applications), adult-only models, non-otologic simulations, and abstracts that lacked full-text versions. Reviews that did not include original data were also excluded.

### 2.3. Study Selection

After removal of duplicates, two reviewers (S.C. and C.C.) independently screened titles and abstracts and assessed these for eligibility against the predetermined inclusion criteria. They compared their lists of included and excluded studies. The full texts of selected articles were reviewed according to eligibility criteria. Any discrepancies arising during this process were resolved through discussion and consensus, or by consulting a third author (E.C.). A standardized data extraction sheet was used to collect study characteristics (author, year, country, study design), model type (3DP, VR, cadaveric, animal), imaging source and manufacturing/technical details, number of participants, number of models/cases, surgical procedure(s) simulated, outcomes assessed (surgical training, preoperative rehearsal, anatomic assessment), and evaluation methods. Due to heterogeneity in study design, model construction, and outcome measures, a descriptive synthesis was performed. Studies were grouped by model type (3DP, VR, cadaveric, animal). Quantitative pooling (meta-analysis) was not planned.

## 3. Results

### 3.1. Pediatric 3DP Models

**Figure 1:**
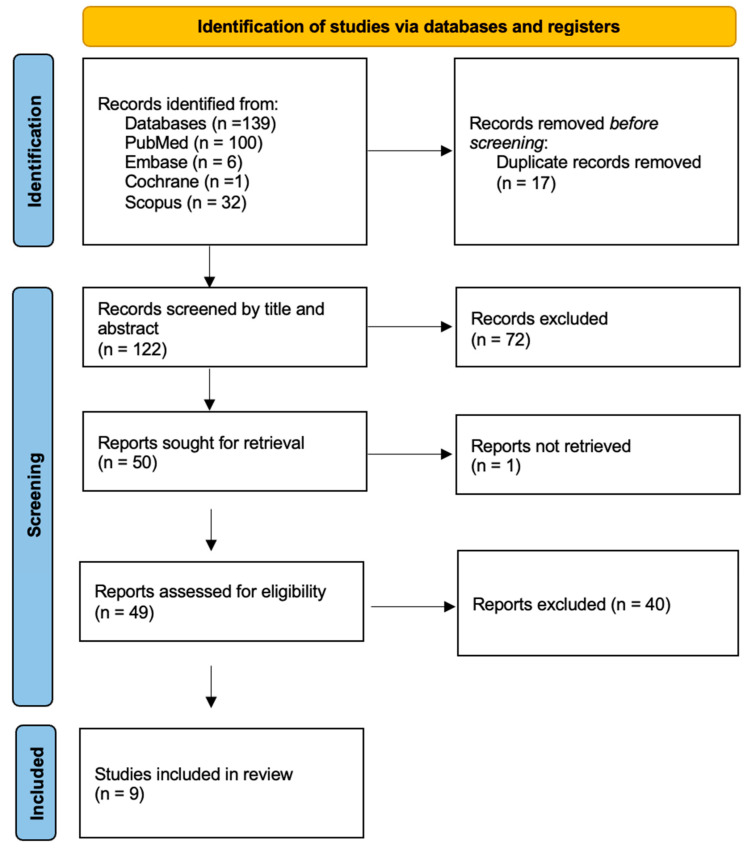
PRISMA flow diagram for pediatric 3DP models.

The search found a total of 139 studies: 100 from PubMed, 6 from Embase, 1 from The Cochrane Library, and 32 from Scopus. After removing duplicates, the total number of articles decreased to 122. After the screening by title and abstract and the removal of 1 article not retrieved, the full texts of the remaining 49 articles were reviewed. Forty studies were excluded from the analysis because they did not meet the inclusion criteria, resulting in nine studies for the final review. The description of non-pediatric models was the primary reason for exclusion; the remaining studies were excluded because of inappropriate intervention, outcome, and setting. The process of study selection is illustrated in a PRISMA flow diagram (Figure 1).

The studies [6,7,8,9,10,11,12,13,14] which met the inclusion criteria are described in Table 1.

The selected articles were published between 2015 and 2024 and included seven non-comparative educational studies, one case report, and one case series. The models described were derived from radiological data from at least eight children aged between 6 months and 17 years. A total of 113 participants were reported to have trained using these models. Participants were heterogeneous, ranging from experienced surgeons to residents, fellows, and attending surgeons; in one study, medical students were also included [6]. Most of the articles focused on microscopic technique (seven studies: [1,2,3,4,5,8,14]), with authors primarily assessing the adequacy of the 3DP models in supporting early surgical training and improving preoperative planning. On the other hand, two studies specifically addressed endoscopic ear surgery [6,7]. Validation methods were heterogeneous across studies, with most employing survey-based approaches (n = 5). These analyses [6,9,10,11,12] primarily relied on Likert-scale questionnaires, in which participants rated anatomical realism and overall simulation experience. Taken together, these studies reported high satisfaction, perceived realism, and improved anatomical understanding. In contrast, three studies [7,8,14] incorporated expert evaluation by otologic surgeons, who qualitatively assessed performance and model fidelity. In the experience reported by Longfield et al., the models were judged to be accurate and clinically informative [8]. Rose et al. assessed model accuracy by measuring the correspondence of distances between anatomical landmarks in 3DP models, CT imaging, and intraoperative findings [7]. Rienas et al. provided indirect validation, suggesting that these models may support preoperative planning and reduce surgical time [14]. One study did not include a formal validation process [13].

The technical aspects of 3DP model production are shown in Table 2.

### 3.2. Pediatric Virtual Models

**Figure 2:**
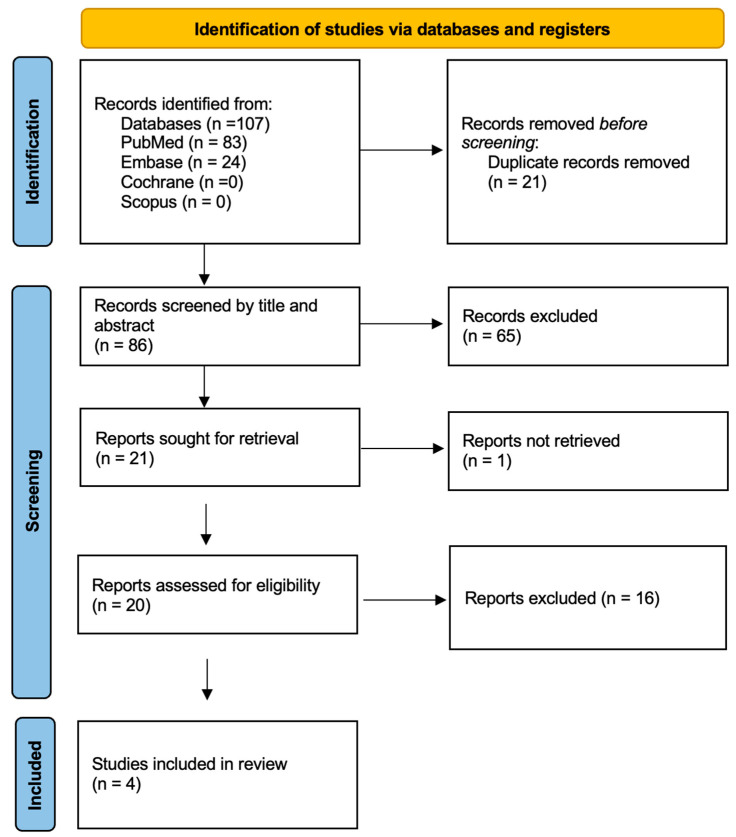
PRISMA flow diagram for VR models.

As a result of the literature search, 107 studies were identified in the first stage. After evaluation of the abstracts and exclusion of out-of-topic studies, the full texts of 20 papers were collected and examined. In only one case the full text was not available. Sixteen studies were excluded from the analysis because they did not meet the inclusion criteria, resulting in four studies for the final review. The PRISMA flowchart regarding the selection process is presented in Figure 2.

The studies [15,16,17,18] which met the inclusion criteria are described in Table 3.

The selected articles were four educational (simulation-based) studies published between 2006 and 2022. VR models identified in this review fell into two categories. The first one [15,17,18] consisted of anatomically realistic environments designed to accurately reproduce pediatric temporal bone structures for anatomical education or surgical training. These patient-derived virtual models were obtained with different methods. Andersen et al. described segmentation of clinical CT imaging studies in children aged from three months to twelve years with normal temporal bone anatomy, enabling the evaluation of patient-specific surgical simulations [17]. Guigou and colleagues developed three pediatric temporal bone resin models (of children aged 1, 2.5, and 6 years, respectively) used as the base for augmented reality-guided systems [18]. Wang et al. obtained a virtual model of a temporal bone based on serial histologic sections from a 14-year-old donor [15]. In the other category, Barber and colleagues described a non-anatomic virtual environment developed primarily to train specific psychomotor skills rather than finely replicate anatomy [16]. The authors developed a testing box that simulates the tympanic cavity. They used computer-aided design software and anatomic measurements from anthropometric studies to create it. This was not exactly a virtual model but rather a modular testing platform for transcanal endoscopic ear surgery. All the above-mentioned experiences aim first to improve anatomical knowledge, or to provide controlled, repeatable platforms for acquiring technical skills. No authors proposed validation of the educational tools through survey-based methods; instead, different quantitative approaches were adopted [16,17,18]. Barber et al. described tool validation through participant-based testing [16]. Andersen et al. presented a feasibility study on various methods for segmenting pediatric temporal bone structures, including a quantitative comparison of segmentation times and volumes across techniques [17]. Additionally, Guigou et al. evaluated task outcomes using post-procedural CT imaging [18]. On the other hand, the study by Wang et al. was purely a feasibility assessment of model development and did not include surgical validation or human participant testing [15]. Despite the variability in evaluation methods, all VR models proved to be valid and reliable for their intended purpose. The technical characteristics of the models described in the selected works are shown in Table 4.

### 3.3. Pediatric Cadaver Models

Of the 29 records identified and screened by title and abstract (5 from PubMed and 24 from Embase), only 1 article was selected for a full-text review. This study was ultimately excluded because, although it was pediatric, it did not focus on otologic surgical simulation. No additional eligible records were found in the Cochrane Library or Scopus.

### 3.4. Pediatric Animal Models

All 36 of the articles identified in PubMed, Embase, The Cochrane Library, and Scopus were excluded after abstract review: all of them were not pertinent.

## 4. Discussion

Newcomers to pediatric otologic surgery face technical challenges that involve a steep learning curve and limited opportunities for safe practice. The present systematic review aims to provide a comprehensive overview of the existing training tools in this field. Available data reveal that technological advances are playing a pivotal role in otosurgical education. Indeed, all thirteen of the included studies focus on 3DP temporal bone models and VR systems, whereas cadaveric and animal models are not mentioned. This reflects ethical issues in using children’s and puppies’ temporal bone specimens for training. In addition, although animal models have historically been valuable in otosurgery, they have limited relevance to the pediatric field as no species replicates the unique size, morphology, or developmental variability of the human pediatric temporal bone [19]. This raises the issue of finding alternatives that overcome this anatomical inadequacy.

Consistently, 3DP technology enables rapid and cost-effective production of bespoke models with strong geometrical details from digital data. For this reason, 3DP models are well-established tools for surgical training or preoperative planning in otorhinolaryngology [3]. In this respect, they appear to be particularly suitable for addressing this issue in pediatric otosurgical education. Indeed, in the existing study, anatomical fidelity was rated as high for 3DP models, particularly those made with multimaterial photopolymer technologies that can replicate the intricate structures of the middle and inner ear with remarkable realism [6,7,9,11,12]. Trainees often reported that the drilling sensations they experienced, such as resistance, vibration, and heat transmission, resembled those in clinical practice [6,9,10,11,12]. These findings suggest that 3DP models provide an adequate approximation for both microscopic and endoscopic skill acquisition. Overall, the available data support the role of patient-specific 3D printing in training and preoperative rehearsal, especially in complex cases such as congenital anomalies.

VR models consist of engineered and digital platforms that offer reproducibility, safety, and adaptability—essential qualities for training in the context of small and developmentally variable anatomy. Realistic VR platforms enabled detailed spatial exploration, familiarization with ear structures and surgical procedures [15,17,18]. In contrast, non-anatomical VR environments provided controlled settings for developing visuomotor and surgical skills. Only one article described this type of tool, designed for training in endoscopic surgery. This solution offered unlimited repetition at a low cost despite lacking anatomical fidelity [16].

Considering both the article about 3DP models and VR simulators, the profile of participants warrants consideration. Most studies involved early-career trainees, such as medical students or junior residents, who had limited prior exposure to otologic anatomy for both microscopic and endoscopic surgical education [15,16,17,18]. Interestingly, some 3DP, patient-specific models have been proposed for the preoperative rehearsal of expert practitioners in challenging situations, such as congenital anomalies [7,14]. Conversely, none of the VR models were used for patient-specific preoperative training. This does not imply that the latter tools are more useful for early-stage education than for advanced surgical rehearsal. Indeed, Guigou et al. described how an experienced surgeon used an augmented reality system to practice complex procedures such as transmodiolar auditory implantation via the middle ear cavity [18].

In our view, the most significant limitation of the currently available data is the small sample size: at least 11 3DP models (in multiple copies) and 4 VR models. Furthermore, the included studies showed wide heterogeneity in terms of study design, participants, validation methods, and evaluated outcomes. These data combined with the preponderance of non-comparative studies, case reports and single-institution case series restrict the comparability of results and impedes the establishment of standardized validation frameworks. We advocate future works prioritizing standardized validation, multicenter collaboration, and testing in broad cohorts of models with normal and malformed anatomies.

Another aspect worth discussing is the clear predominance of U.S.-based studies [6,7,8,10,11,12,13,14,15,16,17]. In fact, 3DP and VR processes entail an interdisciplinary approach involving close collaboration between surgeons, biomedical engineers, and additive-manufacturing specialists. In this respect, we can speculate that these technologies are concentrated in settings with dedicated engineering support and access to advanced fabrication facilities. We believe that providing institutions dedicated to clinical teaching with shared access to 3DP and VR platforms could increase the availability of surgical simulation models. Such an investment would be used in various fields of surgical education, not just in the specific area of pediatric otosurgery. In addition to this, it would reduce collateral costs; for example, practicing with these models does not require equipped and authorized laboratories. Furthermore, in addition to educational aspects, it could have significant clinical implications. Pediatric cases involving congenital malformations or rare conditions are often challenging and may raise concerns even among experienced surgeons. The conversion of radiological data—including temporal bone CT, brain MRI, and, when indicated, MR and CT angiography—into patient-specific models enables detailed preoperative planning and surgical rehearsal, thereby reducing operating time and risk.

## 5. Conclusions

Simulation-based strategies, particularly 3DP pediatric temporal bone models and VR platforms, offer a promising solution to the limited cadaveric or animal resources and the technical difficulties associated with pediatric otologic training. Available evidence suggests that these simulation technologies are valuable tools, particularly as an initial step in an integrated, multimodal surgical training curriculum. Nevertheless, they could also be useful for experienced pediatric otosurgeons when planning particularly complex procedures or experimenting with unusual techniques.

## Figures and Tables

**Table 1:** Pediatric 3DP models.

	Authors, Year	Country	Study Design	N° of Models	N° 3DP Copies	Participants	Technique	Outcomes	Validation Method
1	Ospina et al. 2019 [6]	USA, Colombia	ES	NA	NA	NA	Microscopic surgery (drilling dissection)	Surgical training	Survey
2	Rose et al. 2015 [7]	USA	CR	1 (11 y.o., prior mastoid surgery)	2	2 attendings	Microscopic surgery (tympanoplasty)	Preoperative rehearsal	Experienced surgeon assessment
3	Longfield et al. 2015 [8]	USA	ES	1 (6-mo.o., normal)	3	1 otologic surgeon	Microscopic surgery (mastoidectomy, posterior tympanotomy, cochleostomy)	Surgical training	Experienced surgeon assessment
4	Probst et al. 2018 [9]	Switzerland, Germany	ES	1 (1 y.o., normal)	NA	15 experienced surgeons	Microscopic surgery (cochlear implantation)	Surgical training	Survey
5	Freiser et al. 2021 [10]	USA	ES	1 (7 y.o., normal)	12	4 experienced surgeons, 4 old residents, 4 young residents	Microscopic surgery (mastoidectomy with facial recess approach)	Surgical training	Survey
6	Jenks et al. 2021 [11]	USA	ES	1, NA (normal)	1 NA	32 students and 14 residents	Endoscopic technique (identification of middle ear structures)	Surgical training	Survey
7	Stramiello et al. 2022 [12]	USA	ES	1 (7 y.o., normal)	24	24 (19 residents, 1 fellow, 4 attendings)	Endoscopic technique (identification of middle ear structures)	Surgical training	Survey
8	Freiser et al. 2024 [13]	USA	ES	1 (7 y.o., normal)	7	7 (3 experienced surgeons, 2 fellows, and 2 residents)	Microscopic surgery (mastoidectomy with facial recess approach)	Surgical training	None
9	Rienas et al. 2024 [14]	USA	CS	3 (5 y.o., bilateral absent modioli; 7 y.o., displaced facial nerve; 17 y.o., mastoid cholesteatoma and SC fistulas)	1 for each model	NA	Microscopic surgery (cochlear implantation)	Preoperative rehearsal	Experienced surgeon assessment

N° of model: refers to the patients from whom the data for the 3DP models was derived. The patients’ ages and the features of their temporal bone anatomy are specified. N° 3DP copies refers to the number of printed models from patient-specific data. Outcomes specify whether validation targeted trainee education or preoperative surgical rehearsal. mo.o.: months old; N°: number; NA: not available; y.o.: years old; ES = educational study; CS = case series; CR = case report.

**Table 2:** Technical features of 3DP model production.

	Authors	Year	Software	Materials	Printer
1	Ospina et al. [6]	2019	3D Slicer + Blender + Meshmixer Autodesk	Photopolymer	Fused deposition modeling printer (Fused Form)
2	Rose et al. [7]	2015	Mimics Innovation Suite, Materialise	Photopolymer	Objet350 Connex printer (Stratasys)
3	Longfield et al. [8]	2015	NA	Plaster	Z-Corp Spectrum Z510 printer
4	Probst et al. [9]	2018	Proprietary PHACON workflow	Photopolymer	ZTM510 4D Concepts printer
5	Freiser et al. [10]	2021	3D Slicer + Meshmixer (Autodesk Inc.) + Blender (Blender Foundation)	Acrylic resin	Form Labs 2 stereolithography printer
6	Jenks et al. [11]	2021	Materialise Mimic + Pixologic ZBrush + Maxon Cinema 4D R21	Photopolymer	J750 Digital Anatomy Printer, Stratasys
7	Stramiello et al. [12]	2022	Materialise Mimic	Photopolymer	J750 Digital Anatomy Printer, Stratasys
8	Freiser et al. [13]	2024	3D Slicer + Meshmixer (Autodesk Inc.) + Blender (Blender Foundation)	Acrylic resin	Formlabs Form 2 stereolithography printer
9	Rienas et al. [14]	2024	Materialise Mimics	Resin-based multimaterial	Projet 5500×-E printer

Software refers to the software used to convert the computed tomography images into a format suitable for 3DP. Materials refers to the material used for the 3DP models. NA: not available.

**Table 3:** Pediatric virtual models.

	Authors, Year	Country	Study Design	Data Source	Participants	Techniques	Outcomes	Validation Method
1	Wang et al. 2006 [15]	USA	ES	1 (14 y.o., normal)	NA	NA	Anatomic assessment	NA
2	Barber et al. 2016 [16]	USA	ES	NA	6 residents/otology fellows	Endoscopic technique (transcanal simulated middle ear approach)	Surgical training	Measuring task completion time across repeated simulator trials
3	Andersen et al. 2021 [17]	USA	ES	9 (3 m.o. to 12 y.o., normal)	2 clinicians (for manual segmentation)	NA	Anatomic assessment	Quantitative comparisons of segmentation times and volumes
4	Guigou et al. 2022 [18]	France	ES	3 NA	1 experienced otologic surgeon	Microscopic surgery (transmodiolar auditory implantation via the middle ear cavity)	Surgical training	Postoperative CT image-based error analysis

Data source refers to the patients from whom the data for the VR models was derived. Patient age and temporal bone anatomy features are specified. NA: not available; ES = educational study.

**Table 4:** Technical features of pediatric virtual models.

	Authors	Year	Methods	Software	Image Source
1	Wang et al. [15]	2006	Alignment of digitalized two-dimensional sections, extraction of key anatomic structures, generation of polygonal models	Amira 3.1 for segmentation and 3D reconstruction; 3-D Surface Viewer to display the 3D models	Serial histologic sections of a 14-year-old human temporal bone
2	Barber et al. [16]	2016	Modified tympanic cavity simulator designed in computer-aided design (CAD) software using anatomic measurements from anthropometric studies	Autodesk 123D for Computer-Assisted Drawing, Sculpteo for 3DP	Cross-sectional images of the external auditory canal serially combined into a mesh construct
3	Andersen et al. [17]	2021	Different approaches to segmentation of temporal bone key structures: manual, OTOPLAN-guided, atlas-based automated segmentation	Fiji for image processing, ITK-SNAP for manual segmentation, OTOPLAN^®^ for guided segmentation, OSU atlas for image orientation, Elastix for automated segmentation, Python and SPSS for image comparison and analysis	Clinical CT images
4	Guigou et al. [18]	2022	Temporal bone resin phantoms implanted through navigational augmented reality system	Osirix MPR for model creation, Unity (for preplanning software for augmented navigational information), SURF for motion tracking	Clinical CT images

Software represents the software used for VR system creation.

## Data Availability

The original contributions presented in this study are included in the article. Further inquiries can be directed to the corresponding author.

## Supplementary Materials

The following supporting information can be downloaded at: https://www.mdpi.com/article/10.3390/children13040562/s1, Checklist item.
