# Supplementary material for: Models for Training in Pediatric Otologic Surgery: A Systematic Review
Source: Children (Basel). 2026 Apr 18;13(4):562. doi: 10.3390/children13040562 (PMC13114958; doi:10.3390/children13040562)
Supplement: Supplementary file 1 [file children-13-00562-s001.zip › children-4237215-supplementary.pdf]

PRISMA 2020 Checklist

Section and Topic	Item #	Checklist item	Location where item is reported
**TITLE**			
Title	1	Models for training in Pediatric Otologic Surgery: A Systematic Review	1
**ABSTRACT**			
Abstract	2	Background/Objectives: Temporal bone surgery in children is technically challenging due to their smaller anatomy, developmental differences and the closer proximity of critical neurovascular structures. The limited availability of conventional training materials and pediatric cadaver samples has led to greater enthusiasm for simulation-based methods. The aim of this systematic review was to identify existing otologic simulation models and evaluate their anatomical accuracy, teaching effectiveness and supporting evidence.<br><br>Methods: In accordance with PRISMA guidelines, the PubMed, Embase, Scopus and Cochrane Library databases were searched for studies reporting simulation tools for pediatric otologic surgery. Articles describing three-dimensional printed (3DP) models, virtual reality platforms, cadaver specimens, and animal models were included. The studies focusing on children and providing educational outcomes were selected. The gathered data were synthetized analytically discussed.<br><br>Results: Thirteen studies met the inclusion criteria: nine for 3DP models and four for virtual reality (VR) environments. No research involving cadavers or animals was identified. 3DP models exhibited consistent precision and notable educational advantages. Six studies used surveys for their evaluations, and three relied on assessments by expert observers. Some studies provided validation, revealing a high correlation between printed anatomy and computed tomography (CT) scans images. VR systems supported anatomical reconstruction and segmentation tasks, as well as guided simulation exercises. However, most of the research consisted of feasibility studies with small participant groups.<br><br>Conclusions: Simulation-based training with 3DP and VR models could be ethical and accurate methods for obtaining relevant skills in pediatric otologic surgery. Reviewed data suggest that these tools may be appropriate as first-line step in an integrated and multimodal training prior to direct patient contact.	1
**INTRODUCTION**			
Rationale	3	Temporal bone surgery in children presents technical challenges compared with adult otologic surgery. Literature has demonstrated a growing interest in various simulation-based training models, such as three-dimensional printed (3DP) and virtual reality (VR) tools, in many fields. However, evidence regarding their diffusion, technical characteristics, clinical relevance, and validation within pediatric otologic training remains fragmented across heterogeneous studies.	2
Objectives	4	(1) identify and categorize the types of models described in the literature;<br>(2) assess reported outcomes, including anatomical fidelity, educational effectiveness, and feasibility;<br>(3) map existing gaps to facilitate future research and guide the development of pediatric-specific training tools.	1
**METHODS**			
Eligibility criteria	5	Original studies on pediatric temporal bone surgical training using alternatively 3DP, VR, cadaveric, or animal-besed models; focus on pediatric-specific models or models that were explicitly used for pediatric cases. They had to report educational, anatomical or technical outcomes. Only	2

Section and Topic	Item #	Checklist item	Location where item is reported
		articles published in English were considered.<br>The exclusion criteria were non-English articles, non-surgical studies, adult-only models, non-otologic simulations, and abstracts that lacked full-text versions.	
Information sources	6	PubMed, Embase, The Cochrane Library and Scopus (last consultation: November 2025)	2
Search strategy	7	3D PRINTED MODELS:<br>-PUBMED: ("Temporal Bone"[MeSH] OR "petrous bone" OR "petrous apex" OR "petrous portion" OR "temporal bone") AND ("Three-Dimensional Imaging"[MeSH] OR "Printing, Three-Dimensional"[MeSH] OR "Models, Anatomic"[MeSH] OR "3D model*" OR "three-dimensional model*" OR "3D reconstruction" OR "virtual model*" OR "3D printing" OR "stereolithography" OR "3D simulation" OR "3D visualization")AND ("Child"[MeSH] OR "Infant"[MeSH] OR "Adolescent"[MeSH] OR child* OR pediatric* OR infant* OR newborn* OR neonate* OR adolescent* OR youth)<br>-EMBASE: ('temporal bone'/exp OR 'petrous bone':ti,ab OR 'petrous apex':ti,ab OR 'petrous portion':ti,ab OR 'temporal bone':ti,ab) AND ('three dimensional imaging'/exp OR 'three-dimensional printing'/exp OR 'anatomical model'/exp OR '3d model*':ti,ab OR 'three-dimensional model*':ti,ab OR '3d reconstruction':ti,ab OR 'virtual model*':ti,ab OR '3d printing':ti,ab OR stereolithography:ti,ab OR '3d simulation':ti,ab OR '3d visualization':ti,ab) AND ('pediatrics'/exp OR 'child*':ti,ab OR 'pediatric*':ti,ab OR 'infant*':ti,ab OR 'newborn*':ti,ab OR 'neonate*':ti,ab OR 'adolescent*':ti,ab OR 'youth':ti,ab)<br>-THE COCHRANE LIBRARY: ([MeSH Temporal Bone] OR "petrous bone" OR "petrous apex" OR "petrous portion" OR "temporal bone") AND ([MeSH Three-Dimensional Imaging] OR [MeSH Printing, Three-Dimensional] OR [MeSH Models, Anatomic] OR "3D model*" OR "three-dimensional model*" OR "3D reconstruction" OR "virtual model*" OR "3D printing" OR stereolithography OR "3D simulation" OR "3D visualization") AND ([MeSH Child] OR [MeSH Infant] OR [MeSH Adolescent] OR child* OR pediatric* OR infant* OR newborn* OR neonate* OR adolescent* OR youth)<br>-SCOPUS: (TITLE-ABS-KEY("temporal bone" OR "petrous bone" OR "petrous apex" OR "petrous portion")) AND (TITLE-ABS-KEY("three-dimensional imaging" OR "three-dimensional printing" OR "anatomic model" OR "3D model*" OR "three-dimensional model*" OR "3D reconstruction" OR "virtual model*" OR "3D printing" OR stereolithography OR "3D simulation" OR "3D visualization")) AND (TITLE-ABS-KEY(pediatric* OR child* OR infant* OR newborn* OR neonate* OR adolescent* OR youth))<br><br>VR MODELS:<br>-PUBMED: ("Temporal Bone"[MeSH] OR "Ear, Middle"[MeSH] OR "Skull Base"[MeSH]) AND ("Models, Anatomic"[MeSH] OR "Computer Simulation"[MeSH] OR "Virtual Reality"[MeSH] OR "Simulation Training"[MeSH]) AND ("Pediatrics"[MeSH] OR pediatric* OR child* OR infant* OR neonate* OR adolescent*) AND (surg* OR dissection OR "Surgery, Computer-Assisted"[MeSH])<br>-EMBASE: ('temporal bone'/exp OR 'middle ear'/exp OR 'skull base'/exp) AND ('anatomical model'/exp OR 'computer simulation'/exp OR 'virtual reality'/exp OR 'simulation training'/exp) AND ('pediatrics'/exp OR pediatric*:ti,ab OR child*:ti,ab OR infant*:ti,ab OR neonate*:ti,ab OR adolescent*:ti,ab) AND (surg*:ti,ab OR dissection:ti,ab OR 'computer assisted surgery'/exp)<br>-THE COCHRANE LIBRARY: ([MeSH Temporal Bone] OR [MeSH Ear, Middle] OR [MeSH Skull Base]) AND ([MeSH Models, Anatomic] OR [MeSH Computer Simulation] OR [MeSH Virtual Reality] OR [MeSH Simulation Training]) AND ([MeSH Pediatrics] OR pediatric* OR child* OR infant* OR neonate* OR adolescent*) AND (surg* OR dissection OR [MeSH Surgery, Computer-Assisted])<br>-SCOPUS: (TITLE-ABS-KEY("temporal bone" OR "middle ear" OR "skull base")) AND (TITLE-ABS-KEY("anatomic model" OR "anatomical model" OR "3d model" OR "virtual reality" OR "computer simulation" OR "simulation training")) AND	2

Section and Topic	Item #	Checklist item	Location where item is reported
		TITLE-ABS-KEY(pediatric* OR child* OR infant* OR neonate* OR adolescent*)) AND (TITLE-ABS-KEY(surg* OR dissection OR "computer assisted surgery"))<br><br>CADAVER MODELS<br>-PUBMED: ("Temporal Bone"[Mesh] OR "Ear, Middle"[Mesh]) AND ("Cadaver"[Mesh] OR cadaver*[tiab] OR "Anatomy, Cross-Sectional"[Mesh]) AND ("Pediatrics"[Mesh] OR pediatric*[tiab] OR child*[tiab] OR infant*[tiab]) AND (temporal bone surgery[tiab] OR mastoidectomy[tiab] OR cochlear implant*[tiab] OR otologic[tiab] OR ear surgery[tiab] OR "Otorhinolaryngologic Surgical Procedures"[Mesh]) AND (simulation[tiab] OR training[tiab] OR dissection[tiab])<br>-EMBASE: ('temporal bone'/exp OR 'middle ear'/exp OR otologic*:ab,ti) AND ('cadaver'/exp OR cadaver*:ab,ti OR 'anatomic dissection'/exp) AND ('pediatrics'/exp OR pediatric*:ab,ti OR child*:ab,ti OR infant*:ab,ti OR neonatal*:ab,ti) AND (surg*:ab,ti OR dissection:ab,ti OR 'surgical simulation'/exp OR simulation:ab,ti)<br>-THE COCHRANE LIBRARY: ("temporal bone" OR "middle ear" OR otologic*) AND (cadaver OR cadaveric OR "anatomical dissection") AND (pediatric OR child OR infant OR neonatal) AND (surgery OR dissection OR "surgical training" OR simulation)<br>-SCOPUS: (TITLE-ABS-KEY("temporal bone" OR "middle ear" OR otologic*)) AND (TITLE-ABS-KEY(cadaver* OR "cadaveric dissection" OR "anatomy dissection")) AND (TITLE-ABS-KEY(pediatric* OR child* OR infant* OR neonatal*)) AND (TITLE-ABS-KEY(surg* OR dissection OR "surgical training" OR simulation OR "surgical simulation"))<br><br>ANIMAL MODELS<br>-PUBMED: ("Temporal Bone"[Mesh] OR "Ear, Middle"[Mesh]) AND ("Animal Experimentation"[Mesh] OR "Animal Models"[Mesh] OR animal*[tiab] OR ovine[tiab] OR porcine[tiab] OR piglet[tiab] OR lamb[tiab] OR rabbit[tiab]) AND (pediatric*[tiab] OR child*[tiab] OR infant*[tiab] OR newborn*[tiab]) AND (temporal bone surgery[tiab] OR mastoidectomy[tiab] OR otologic[tiab] OR cochlear implant*[tiab]) AND (simulation[tiab] OR training[tiab] OR surgical model[tiab] OR dissection[tiab])<br>-EMBASE: ('temporal bone'/exp OR 'middle ear'/exp OR otologic*:ab,ti) AND ('animal experiment'/exp OR 'animal model'/exp OR porcine:ab,ti OR ovine:ab,ti OR sheep:ab,ti OR pig:ab,ti OR rabbit:ab,ti) AND ('pediatrics'/exp OR pediatric*:ab,ti OR child*:ab,ti OR infant*:ab,ti OR neonatal*:ab,ti) AND (surg*:ab,ti OR dissection:ab,ti OR 'surgical simulation'/exp OR simulation:ab,ti) NOT (cochlear:ab,ti AND (physiology:ab,ti OR neuron*:ab,ti OR 'inferior colliculus':ab,ti))<br>-THE COCHRANE LIBRARY: ("temporal bone" OR "middle ear" OR otologic*) AND (animal OR "animal model" OR porcine OR pig OR ovine OR sheep OR rabbit) AND (pediatric OR child OR infant OR neonatal) AND (surgery OR dissection OR "surgical training" OR simulation)<br>-SCOPUS: (TITLE-ABS-KEY("temporal bone" OR "middle ear" OR otologic* OR otolog*)) AND (TITLE-ABS-KEY(animal* OR "animal model*" OR porcine OR ovine OR sheep OR pig OR rabbit)) AND (TITLE-ABS-KEY(pediatric* OR child* OR infant* OR neonatal*)) AND (TITLE-ABS-KEY(surg* OR dissection OR "surgical training" OR simulation OR "surgical simulation")) AND NOT TITLE-ABS-KEY(cochlear AND (physiology OR neuron* OR inferior colliculus OR ITD))	
Selection process	8	Two reviewers independently screened records and full texts. Disagreements were resolved by consensus or a third reviewer (E.C.). No automation tools were used.	2
Data collection process	9	Two reviewers independently screened records and full texts. Disagreements were resolved by consensus or a third reviewer (E.C.). No automation tools were used.	2
Data items	10a	Data were extracted on model type (3DP, VR, cadaveric, animal)	2

PRISMA

PRISMA 2020 Checklist

Section and Topic	Item #	Checklist item	Location where item is reported
	10b	Study characteristics (author, year, country), model type (3DP, VR, cadaveric, animal), imaging source, technical/manufacturing details, number of participants and cases, surgical procedures, and evaluation methods. Outcomes included surgical training, pre-operative rehearsal, and anatomical assessment. All relevant reported results for these outcomes were collected where available. In cases of missing or unclear data, assumptions were made based on the information provided in the study.	3
Study risk of bias assessment	11	Risk of bias was assessed independently by two reviewers using an appropriate standardized tool. Discrepancies were resolved by consensus or by consultation with a third reviewer (E.C.). No automation tools were used.	3
Effect measures	12	Not applicable.	NA
Synthesis methods	13a	Studies were grouped by model type (3DP, VR, cadaveric, animal) and compared according to the predefined outcomes.	2
	13b	No data conversions were required; available data were extracted as reported. Missing or unclear data were addressed descriptively where possible.	NA
	13c	Results were summarized in tables and described narratively.	4-10
	13d	Due to heterogeneity in study design, interventions, and outcomes, a descriptive (narrative) synthesis was performed; meta-analysis was not conducted.	4-10
	13e	No formal analyses of heterogeneity (e.g. subgroup analysis or meta-regression) were performed.	NA
	13f	No sensitivity analyses were conducted.	NA
Reporting bias assessment	14	Risk of bias due to missing results (reporting bias) was not formally assessed.	NA
Certainty assessment	15	The certainty of the evidence was not formally evaluated.	NA
**RESULTS**			
Study selection	16a	The search identified 139 records for 3DP models and 107 for VR models. After duplicate removal and screening, 9 studies on pediatric 3DP models and 4 on VR models were included. No eligible studies were identified for cadaveric or animal models. The study selection process is presented in the flow diagrams	3
	16b	Studies excluded after full-text review did not meet inclusion criteria, mainly due to non-pediatric populations, inappropriate intervention, outcomes, or setting.	3
Study characteristics	17	Characteristics of included studies are reported in Tables 1–4, including study design, population, model type, techniques, and outcomes.	4-10
Risk of bias in studies	18	Risk of bias assessments for included studies are presented in the corresponding tables.	4-10
Results of individual studies	19	Individual study results are summarized descriptively in tables, including study characteristics, validation methods, and reported outcomes.	4-10

Section and Topic	Item #	Checklist item	Location where item is reported
Results of syntheses	20a	Included studies were heterogeneous in design, model construction, and outcome assessment. Most studies reported qualitative or semi-quantitative validation with generally positive findings.	4-10
	20b	No statistical synthesis was performed due to heterogeneity.	NA
	20c	No formal analyses of heterogeneity were conducted.	NA
	20d	No sensitivity analyses were performed.	NA
Reporting biases	21	Reporting bias was not formally assessed.	NA
Certainty of evidence	22	The certainty of the evidence was not formally evaluated.	NA
**DISCUSSION**			
Discussion	23a	This systematic review highlights the increasing role of simulation-based technologies, particularly three-dimensional printing (3DP) and virtual reality (VR), in pediatric otologic training. Compared with traditional cadaveric and animal models—limited by ethical concerns and anatomical differences—these approaches provide reproducible, safe, and anatomically relevant alternatives. Overall, the included studies suggest that 3DP models offer high anatomical fidelity and realistic haptic feedback, while VR platforms provide flexible environments for skill acquisition. These findings are consistent with the growing body of evidence supporting simulation-based training in surgical education.	10-12
	23b	The evidence included in this review is limited by small sample sizes, heterogeneity in study design, variability in validation methods, and frequent reliance on subjective assessments. Most studies were conducted in single centers and primarily involved early-stage trainees, which may limit generalizability. Furthermore, the lack of standardized validation frameworks restricts comparability across studies.	10-12
	23c	This review has some limitations. The search was restricted to selected databases, and potentially relevant studies may have been missed. In addition, the exclusion of non-English or unpublished studies may have introduced selection bias. Due to heterogeneity among studies, quantitative synthesis was not feasible.	12
	23d	Simulation-based tools, particularly 3DP and VR models, represent valuable adjuncts in pediatric otologic training and may help overcome current educational and ethical limitations. Their use may be beneficial both for early-stage training and for preoperative planning in complex cases. Future research should focus on multicenter studies, larger sample sizes, standardized validation methods, and cost-effectiveness analyses to support broader implementation.	12
OTHER INFORMATION			
Registration and protocol	24a	The review was not registered.	NA
	24b	No review protocol was prepared.	NA
	24c	Not applicable.	NA
Support	25	No specific funding was received for this study. The authors declare no role of funders in the design, conduct, or reporting of the review.	NA
Competing interests	26	The authors declare no competing interests.	NA

PRISMA 2020 Checklist

Section and Topic	Item #	Checklist item	Location where item is reported
Availability of data, code and other materials	27	Data extracted from the included studies are presented within the article. Additional materials are available from the corresponding author upon reasonable request.	NA

From: Page MJ, McKenzie JE, Bossuyt PM, Boutron I, Hoffmann TC, Mulrow CD, et al. The PRISMA 2020 statement: an updated guideline for reporting systematic reviews. BMJ 2021;372:n71. doi: 10.1136/bmj.n71.
This work is licensed under CC BY 4.0. To view a copy of this license, visit https://creativecommons.org/licenses/by/4.0/
